#  A Theory-Based mHealth Intervention (Getting Off) for Methamphetamine-Using Men Who Have Sex With Men: Protocol for a Randomized Controlled Trial

**DOI:** 10.2196/22572

**Published:** 2021-02-22

**Authors:** Cathy J Reback, Jesse B Fletcher, Raymond P Mata

**Affiliations:** 1 Friends Research Institution, Inc Los Angeles, CA United States; 2 UCLA Center for HIV Identification, Prevention, and Treatment Services University of California, Los Angeles Los Angeles, CA United States

**Keywords:** HIV, AIDS, methamphetamine, mHealth, mobile app, ART, mobile phone

## Abstract

**Background:**

Methamphetamine (meth) use among men who have sex with men (MSM) is associated with increased HIV prevalence and transmission and substandard advancement along the HIV prevention and care continuum. Given the growth of mobile health (mHealth) technologies, it is no longer necessary to limit meth treatment options to physical, brick-and-mortar sites, and administration using generic, nontailored content.

**Objective:**

In a 2-arm randomized controlled trial (RCT; N=300), we aim to evaluate the use of an mHealth intervention (Getting Off) to assess the impact and noninferiority of a cross-platform app (developed from a manualized meth treatment intervention) to help MSM reduce meth use and HIV sexual risk behaviors and improve their advancement along the HIV prevention and care continuum (HIV testing, pre-exposure prophylaxis uptake and persistence, and antiretroviral therapy uptake and adherence).

**Methods:**

Participants will be randomized into 2 arms: arm A, with immediate access to the app (immediate delivery: n=150), or arm B, with delayed access to the app after a 30-day period (delayed delivery: n=150). Participants in both arms will use the same Getting Off app and will have 30 days to complete the 24 sessions. Participants will be assessed at the 1-, 2- (delayed delivery arm only), 3-, 6-, and 9-month timepoints to determine observed treatment effects and will be compared with a historical matched sample of participants (n=~600) who received the brick-and-mortar group-based Getting Off intervention.

**Results:**

Recruitment began in January 2019 for phase 1, the formative phase. In January and February 2019, 4 focus groups (N=36) were formed to provide input on the adaptation of the group-based manual intervention to a mobile app. Data collection for phase 2, the RCT, is expected to be completed in January 2023. The final results are anticipated in April 2023.

**Conclusions:**

By creating a culturally responsive mobile app, Getting Off aims to reduce meth use and improve sexual health outcomes among meth-using MSM. The Getting Off app could have significant public health impact by greatly expanding access to effective, affordable, private, culturally competent, and highly scalable meth treatment for MSM.

**Trial Registration:**

Clinicaltrials.gov NCT03884946; https://clinicaltrials.gov/ct2/show/NCT03884946

**International Registered Report Identifier (IRRID):**

DERR1-10.2196/22572

## Introduction

### Background

Men who have sex with men (MSM) have elevated rates of methamphetamine (meth) use relative to non-MSM [1-3], as meth use is deeply integrated into the sexual identities and sexual behaviors of MSM in the United States [3-7] and permeates the venues most often associated with high-risk sexual behaviors among MSM [8-10]. Use of meth by MSM before or during sex is associated with decreased behavioral inhibition and increased engagement in HIV risk behaviors [11-14], including condomless anal intercourse (CAI) [15-22] with serodiscordant or HIV status unknown sexual partners [23-25].

HIV prevalence is significantly higher among MSM who use meth [14,25-29] and increases in concert with the intensity of meth use [30]. Meth use has thus been identified by the Centers for Disease Control and Prevention (CDC) as a driving force of the HIV epidemic among MSM in the United States [13]. Meth use is associated with poor antiretroviral therapy (ART) adherence and outcomes [31-34] and reduced adherence to HIV postexposure prophylaxis (PEP) and pre-exposure prophylaxis (PrEP) among HIV-positive/-negative MSM, respectively [35]. Providing meth treatment to MSM is a public health imperative for addressing HIV/AIDS in the 21st century.

MSM use smartphones for sexual partner selection, sexual health information, and sexual identity expression at a higher rate than non-MSM [36-41], and they also use smartphone apps to facilitate GPS-based sexual partner selection (eg, Scruff, Grindr, and Jack’d) [42-44]. Such behaviors increase the odds of both meth use and HIV sexual risk behavior [45]. Young MSM report using such apps daily [37,39,46], and young, racial minority MSM are simultaneously both the group most at risk for meth use and HIV infection [47] as well as the group most likely to use smartphones [38,39,46]. MSM living in rural areas rely on internet resources and GPS-enabled smartphones to locate sexual partners [48]. High rates of smartphone use by young racial or ethnic minority and rural MSM dovetail cleanly with the current meth treatment and HIV risk reduction deficits evidenced in the United States health care system [38]. Psychosocial factors (eg, stigma) are the primary barriers discouraging MSM from accessing meth treatment [49,50], obstacles obviated through technology-based delivery. Given the severe personal and public health consequences of meth use, the ability to access treatment from a smartphone would eliminate embarrassment, homophobic prejudice, and/or any stigma associated with meth use and/or HIV sexual risk behaviors [50,51].

Providing theory-driven, MSM-specific meth treatment, which integrates HIV risk reduction programming, reminders about HIV testing, PrEP, PEP, and information about HIV care (including ART reminders), will address a range of HIV-related health deficits and address key priorities set by the National Institutes of Health HIV/AIDS research priorities and the National HIV/AIDS Strategy. Furthermore, the overall public health benefits could be tremendous, as meth use has also been associated with major physical harm [52], dental disease [53], psychological harm [15,54-56], and neurological damage [57]. The broader scientific community would benefit substantially from the knowledge that meth use and HIV sexual risk behaviors can successfully be reduced via remote intervention, and clinical practice could face a potential paradigm shift toward mobile content delivery for difficult-to-reach and/or stigmatized populations.

Given the growth of mobile technology, it is no longer reasonable or necessary to limit meth treatment to physical, brick-and-mortar sites. Only 1% of app-using MSM express a preference to participate in programs delivered in person, whereas 70% prefer content delivered via smartphones [36]. The Getting Off intervention is particularly well-situated for translation into a mobile app–driven format, as it has already been adapted to be carried out in community settings with peer counselors and does not require delivery via masters’ level cognitive behavioral therapy (CBT) clinicians [4,58].

Mobile apps are available for download and use 24 hours a day, 7 days a week, making it easier to attempt treatment and access information in a contextualized and need-based manner; such immediacy cannot be achieved at a brick-and-mortar facility. In addition, the adaptation of a group-based intervention to a self-directed and individualized format provides the opportunity for novel delivery modalities such as gamifying formerly group-based intervention activities; insights and information will be conveyed to participants through activities such as sorting, matching, and interactive board games.

### Objectives

The overall goal of this research study is to (1) adapt the Getting Off meth treatment intervention from a physical, brick-and-mortar facility to computerized delivery; a counselor-delivered intervention to self-directed; and a group-based to an individualized format and (2) assess the impact and noninferiority of the Getting Off app. The final Getting Off app will provide meth-using MSM and service providers with a culturally competent, free-to-own, cross-platform mobile health (mHealth) smartphone app that can be broadly disseminated.

## Methods

### Research Aims

The project builds upon the established efficacy of our manualized meth treatment intervention, *Getting Off*: *A Behavioral Treatment Intervention for Gay and Bisexual Male Methamphetamine Users,* and the highly promising findings from our successful stage 1 proof-of-concept study to complete translation of Getting Off into a cross-platform (iOS and Android) mHealth smartphone app and to assess the app’s efficacy *and* noninferiority in a scientifically rigorous randomized trial. The stage 2 development of the cross-platform Getting Off app will be based on user feedback from the feasibility pilot test, current literature on app preferences among MSM, and state-of-the-art technology. The app will be an interactive presentation of the Getting Off manual. Health content, behavioral self-assessments, *homework* assignments, and multimedia content will be integrated in a walk-through (ie, step-by-step) manner, where the consumer will be presented dynamic content, all under the guidance of a culturally competent, user-friendly, and attractive interface. The app could broadly disseminate culturally competent meth use and HIV risk reduction content to large numbers of demographically and geographically diverse users who otherwise could not access such services, particularly racial or ethnic minority MSM and MSM living in rural areas [38,47,59].

The aims of the research include the following:

- Primary aim 1: Refine and enhance the first 8 sessions of the Getting Off meth-use treatment intervention developed in stage 1 based on feasibility pilot test user feedback.
  Approach: Integrate findings from stage 1, and refine or enhance the first 8 sessions with the technology team and input from our community advisory board.
- Primary aim 2: Conduct formative research to develop the remaining 16 sessions of the Getting Off meth-use treatment intervention into a cross-platform computerized mobile app targeted to reduce meth use and HIV sexual risk behaviors and increase advancement along the HIV prevention or care continuum.
  Approach: Design and develop the complete app by guiding technology team revisions using focus group and community advisory board feedback; conduct a usability pilot study to assess the feasibility, acceptability, and preliminary effects of the Getting Off app; and refine app iterations through community input.
- Primary aim 3: Conduct a randomized controlled trial (RCT) to evaluate reductions in meth use and HIV sexual risk behaviors (eg, CAI, engagement in sex work, and sex while feeling the effects of alcohol or drugs) and increase advancement along the HIV prevention (repeat HIV testing, PEP or PrEP linkage and uptake, PEP adherence, and PrEP adherence and persistence) or care continuum (linkage to HIV care, ART adherence, and virological suppression).
  Approach 3a: A 2-arm RCT to determine intervention effects through comparison of the immediate delivery (ID; n=150) and delayed delivery (DD; n=150) arms
    Aim 3a hypothesis: Exposure to the Getting Off app will produce significant reductions in meth use and HIV sexual risk behaviors as well as increased odds of advancement along the HIV prevention or care continuum.
  Approach 3b: An observed treatment effects analysis powered for prospective subgroup contrasts to compare longitudinal pre- or postdata from the pooled ID and DD arms (N=300)
    Aim 3b hypothesis: Exposure to the Getting Off app will be associated with significant reductions in meth use and HIV sexual risk behaviors as well as increased odds of advancement along the HIV prevention or care continuum.
  Approach 3c: A 2-arm historical matched comparison design to evaluate the outcomes of the Getting Off app (ID+DD; N=300) relative to a matched sample of participants who previously attended the brick-and-mortar group-based Getting Off intervention (N=~600; total N=900).
    Aim 3c hypothesis: Reductions in meth use and HIV sexual risk behaviors as well as increased advancement along the HIV prevention or care continuum will be statistically noninferior to those observed in the brick-and-mortar group-based Getting Off intervention.
- Secondary aim 1: Determine the impact of structural- (eg, housing insecurity, food scarcity, educational attainment, and access to health care) and individual-level (eg, homophobia, stigma, and discrimination) factors as moderators of intervention outcomes.

### Ethics Statement

All study procedures were approved by the Western Institutional Review Board (IRB Study #1248891; IRB Tracking #20182737). The study was registered as a clinical trial (Clinical Trials #NCT03884946). All procedures performed in studies involving human participants were in accordance with the ethical standards of the institutional and/or national research committee and with the 1964 Helsinki declaration and its later amendments or comparable ethical standards.

### Study Procedures

#### Aim 1

App refinement of the first 8 sessions addressed the areas identified through the feasibility pilot test. The research and app development teams designed and developed a cross-platform mobile app using a cross-platform framework (eg, Ionic) that will permit downloading the app on both the iOS and Android platforms. The cross-platform framework approach promotes extensive code reusability between the 2 platforms and ensures a consistent user experience. The app will be for the latest versions of iOS and Android operating systems and will include backward compatibility for one operating system version of each.

#### Aim 2

The research team conducted formative research to translate the remaining 16 sessions from the group-based manual-driven intervention to a computerized app, conducted alpha-phase postdevelopment bug testing, conducted beta-phase usability pilot testing, and refined the app according to alpha- and beta-phase testing.

Four focus groups (N=36) provided input on the development of the Getting Off app: (1) out-of-treatment, meth-using MSM (n=10); (2) meth-using MSM who are currently in outpatient treatment in the Getting Off program (n=10); (3) prior meth-using MSM with a minimum of 1 year recovery who have completed a minimum of 18 out of 24 sessions (75%) of the brick-and-mortar Getting Off program (n=6); and (4) prior meth-using MSM with a minimum of 1 year of recovery who have had no previous experience or knowledge of the Getting Off intervention (n=10). The focus groups were structured to provide guidance on translating Getting Off into an app that is responsive to culture (eg, sexuality, HIV prevention or care including PrEP uptake, adherence and persistence, ART adherence, and viral load suppression) and meth treatment needs. Additional input was obtained through the ongoing community advisory board and usability pilot testing.

Following app development, alpha-phase testing has uncovered and removed unwanted bugs. Beta-phase usability pilot testing will be conducted with members of the target population (N=30) to test the feasibility and acceptability of the Getting Off app. Furthermore, pilot testing will be used to ensure the functionality of the app (ie, that the app is user-friendly, and all features function appropriately). Inclusion criteria for the pilot test were as follows: (1) self-identified MSM, (2) prior meth user but no meth use in the past 365 days, (3) aged between 18 and 65 years, and (4) able and willing to provide informed consent. The behavioral assessments will be consistent with those of the RCT. Although the group-based Getting Off intervention is delivered over 8 weeks (24 sessions at 3 groups per week), it is expected that it will take far less time to progress through the self-directed app, and because of the interactive features, participants may choose to replay treatment modules multiple times in the 30-day period. Behavioral assessments will be conducted at baseline and at the 1-month follow-up. In addition, at the 1-month follow-up visit, an open-ended, in-person, qualitative user experience exit interview will be collected to collect feedback on the app, including the perceived benefits, concerns, and suggestions for improvements. Qualitative interviews will be digitally recorded and analyzed using the same methodology as the focus groups.

#### Aim 3

**Figure 1:**
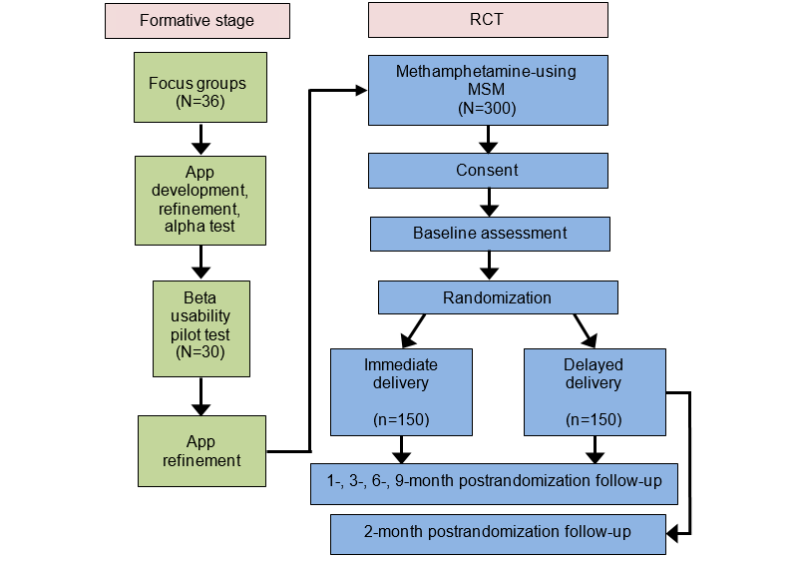
Schematic of study design. MSM: men who have sex with men; RCT: randomized controlled trial.

**Figure 2:**
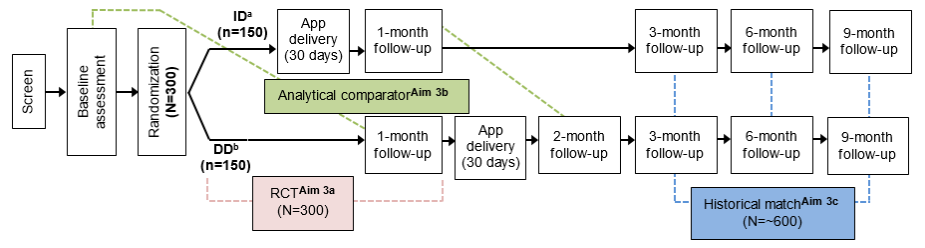
Randomized controlled trial design. DD: delayed delivery; ID: immediate delivery.

Following screening, informed consent, and baseline assessments, participants will be randomized into 1 of 2 arms: arm A, immediate access to the Getting Off app (ID), or arm B, participants will have access to the Getting Off app after a delayed 30-day period (DD). Participants in both arms will receive the same Getting Off app and will be given 30 days to complete the 24 sessions. The randomized 2-arm repeated measures design will assess participants at 1, 2- (DD arm only), 3-, 6-, and 9-months after randomization to determine longitudinal intervention effects, observed treatment effects, and a historical comparison with a matched sample of participants who attended the brick-and-mortar group-based Getting Off intervention (Figures 1 and 2). The study will use an *intent-to-treat* design; participants will be assessed regardless of participation or retention.

### Sample

To ensure comparability of outcomes with those of the group-based Getting Off intervention manual, inclusion criteria must mirror that of the brick-and-mortar treatment site: (1) self-identified MSM, (2) any meth use in the past 365 days, (3) aged between 18 and 65 years, and (4) able and willing to provide informed consent. Both HIV-positive and HIV-negative participants will be eligible.

### Recruitment

To ensure a steady stream of diverse participants, 4 recruitment strategies will be used: (1) online recruitment: banner ads or digital flyers will be placed on gay websites, apps, and social media sites that specifically target MSM, such as Scruff, Adelante, Craigslist, Adam4Adam, Jack’d, and Grindr; established relationships with web-based venues that cater to MSM will enable a successful and robust internet-based recruitment strategy. (2) Street- and venue-based outreach: a semistructured time-space sampling methodology will be used to conduct street- and venue-based outreach identified through the community advisory board and ongoing community mapping as locations where meth-using MSM congregate. (3) Poster advertisement: posters that introduce the study will be posted to inform potential participants who to contact for further information regarding the research study. (4) Long-chain referral sampling: the participants of this study will be asked to recruit potential new participants. The participants of this study participants will receive US $2 when they bring a potential participant to the site and US $18 if an eligible participant is enrolled. Potential participants who inquire about the study will be scheduled for intake within 24-48 hours. These strategies have previously been used to recruit similar samples in prior studies.

### Randomization

Following informed consent and completion of the baseline assessment, participants will be randomized to either the ID or DD arm through a computerized variable-balanced procedure. Recent work with substance-dependent (predominantly meth) MSM revealed treatment outcomes to be associated with participant substance use histories and sociodemographics [60]. A variable-balanced procedure will thus provide a multivariate balance among the characteristics known or expected to influence outcomes. The randomization procedure will balance across age (<34 years or ≥34 years), race or ethnicity (White and all other race or ethnicities), HIV serostatus (+ or −), and stages of change (SOC; contemplation or preparation and action or maintenance) [61].

### Study Arms

The study intervention will be delivered via the identical cross-platform Getting Off mobile app for participants in both arms. Postrandomization participants will download the app onto their phone or tablets. Those randomized into the ID arm will immediately be given a unique user passcode to access the intervention content, whereas those randomized into the DD arm receive their unique user passcode 30 days after randomization (Figure 2).

### Theoretical Foundations: Mechanism of Behavioral Change

**Figure 3:**
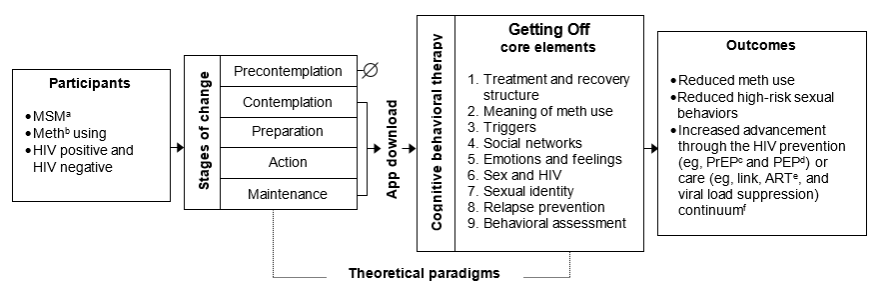
Mechanism of behavior change. ART: antiretroviral therapy; meth: methamphetamine; HIV prevention continuum (HIV testing and pre-exposure prophylaxis/postexposure prophylaxis uptake) and HIV care continuum (link, antiretroviral therapy adherence, and virological suppression); MSM: men who have sex with men; PEP: postexposure prophylaxis; PrEP: pre-exposure prophylaxis.

The SOC model [62-67] conceives of behavior change as a 5-stage process, ranging from not yet considering a particular behavior (ie, precontemplation) through ongoing, long-term maintenance of that same behavior (Figure 3). An individual moves through the stages as they become aware of the need for change, prepare for change, and implement change. Most individuals are in the *action* stage by the time they seek treatment at a brick-and-mortar facility. In contrast, due to the privacy and availability of a computerized intervention, the Getting Off app would be of interest and value to any meth-using MSM who has moved beyond the precontemplation stage, allowing for broader dissemination, earlier interruption of meth use and HIV sexual risk behaviors, and increased opportunities for advancement through the HIV prevention or care continuum, maximizing potential public health impacts.

The computerized Getting Off app, like the group-based intervention before it, will be guided by CBT and will use a broad set of psychological and educational techniques to provide meth-using MSM with critical knowledge about their meth use, teaching skills to initiate abstinence and to return to abstinence should relapse occur [68]. The theoretical principles of CBT have been widely integrated into most interventions for substance use disorders in the United States (including Alcoholics Anonymous) [69] and have shown efficacy for reducing both cocaine and meth use [70-74]. The CBT model in Getting Off provides education on internal and external triggers, stages of recovery from meth use, and identification of emotional states that can signal relapse. The CBT model also teaches cognitive skills such as thought stopping, craving management, relapse analysis, and adoption of healthy lifestyle behaviors. Figure 3 displays the conceptual model of behavior change. The far-left components in the model include the participant profile, the SOC related to meth use that would encourage a MSM to download the Getting Off app, and the core elements of the Getting Off app. Outcomes include reductions in meth use and HIV sexual risk behaviors and increased advancement through the HIV prevention or care continuum.

### Measures

All data will be collected on an Audio Computer-Assisted Self-Interview administered via the Qualtrics system. All materials will be stored in Qualtrics’ Health Insurance Portability and Accountability Act-compliant (secure and encrypted) cloud-based databases. Data from historical comparators will be sampled from participants enrolled in the brick-and-mortar Getting Off intervention beginning in 2012, as research indicates that 2012 is when up to 95% of meth-using MSM in Friends Community Center reported owning their own cellphone [75]. The following subheadings (*Diagnostic Mental Health and Substance Use Disorder*; *Stages of Change*; *Sociodemographics, Familiar, Legal, and Health Status*; *Substance Use*; *Meth Use and Sexual Risks*; and *Biological Markers*) describe the measures used to address the study’s specific aims.

#### Diagnostic Mental Health and Substance Use Disorder

Diagnostic and Statistical Manual of Mental Disorders (DSM-5) Methamphetamine Use Disorder contains the DSM-5 diagnostic items necessary to determine mild, moderate, or severe meth use disorder. This information will determine the app’s utility for consumers at various levels of meth use. It will be administered only at baseline.

#### Stages of Change

The University of Rhode Island Change Assessment (URICA) is a brief, self-administered inventory used to assess the participant’s current position regarding readiness for change (eg, precontemplation, contemplation, and action) [66]. In nontreatment seeking MSM, the participant’s motivation or readiness for change may be an important predictor of response to the computerized intervention app. The URICA will be helpful in characterizing participants’ SOC [76]. It will be administered at all time points.

#### Sociodemographics, Familiar, Legal, and Health Status

Admission or follow-up form collects demographic information, housing status, food security, educational attainment, alcohol and other drug use history, family and social history, legal status, HIV status, location on the HIV prevention or care continuum, experiences with stigma and/or discrimination, and general and mental health status. The full form will be administered at baseline, and an abbreviated version (ie, a version that excludes all “lifetime recalls” and sociodemographic characteristics) will be administered at all follow-up time points.

#### Substance Use

Substance use frequency is a brief assessment, developed by the principal investigator that assesses substance use, injection drug use, and injection protocols in the past 30 days. It will be administered at all time points.

#### Meth Use and Sexual Risks

Behavioral Questionnaire-Amphetamine (BQA)-abbreviated version gathers information on HIV-related drug and sexual risk behaviors, assesses self-efficacy for sexual behavior change, collects detailed information on discrete sexual behaviors (with primary or nonprimary partners and whether or not the behavior occurred under the influence of meth and/or other substances), and collects episodic data about participants’ most recent sexual encounters [77,78]. Although the heterogeneous nature of the material included in the BQA prevents estimation of omnibus reliability coefficients, the assessment has compared favorably in tests against similar instruments and has been validated for use in these and similar populations [70,77-79]. It will be administered at all time points.

#### Biological Markers

Urine drug screen: urine samples will be collected, monitored, and analyzed using a 5-panel Food and Drug Administration–approved urine test cup [80]. The test cup screens for metabolites of the following drugs of use at the noted cut-off levels: amphetamines (1000 ng/mL), cocaine (300 ng/mL), meths (500 ng/mL), opiates (300 ng/mL), and tetrahydrocannabinol (50 ng/mL). Urine sample validity checks will be provided by temperature and adulterant monitoring strips built into the customized test cup. Criteria for validity will be indicated by the temperature of the sample (eg, above 92 degrees Fahrenheit and below 98 degrees Fahrenheit) and the presence of normal ranges of creatinine; pH; specific gravity; and nitrates, pyridinium chlorochromate, and bleach provided by the adulterant strip. Results are coded qualitatively (above or below the threshold) and serve as the primary indicator of recent drug use. It will be administered at all time points.

Rapid HIV antibody test: HIV-negative and status unknown participants will receive a rapid point-of-care fingerstick HIV-antibody blood test [81] at 3-month intervals, as recommended by the CDC for high-risk individuals [82]. Participants who show documentation of HIV-positive serostatus will not be given an HIV antibody test.

Sexually transmitted infection (STI) testing: participants will be tested for *Neisseria gonorrhoeae (N. gonorrhea)* and Chlamydia in the urethra via urine sample. Pharyngeal and rectal swabs will be taken for *N. gonorrhea* and Chlamydia, and syphilis will be tested by serum red plasma reagin and confirmed by fluorescent treponemal antibody absorption testing. Positive STI results will be reported per state guidelines and will be immediately referred to care. Prior studies found high rates of undiagnosed STIs among out-of-treatment, meth-using MSM [29,83], which also serve as a marker of HIV sexual risk behaviors.

Virologic control for HIV-positive participants: as indicated by an undetectable HIV-1 level on the COBAS AmpliPrep/COBAS TaqMan HIV test kit [84], which has a threshold for undetectability set at ≤20 copies/mL, will be performed by Foundation Laboratory. It will be conducted at each 3-month assessment.

Dried blood spot (DBS) for HIV-negative participants who report PrEP uptake: a blood sample will be collected and a DBS analysis for intraerythrocytic tenofovir-diphosphate will be performed. It will be conducted at each 3-month assessment.

Health study locator form: the locator form asks participants to give consent for follow-up and to provide names; addresses; email and internet site profiles, particularly social network sites; and phone numbers of 3 relatives or friends who can reach the participant. Information is also collected on where (libraries, clubs, and bars) and with whom the participant associates (ie, social network). This will be administered at baseline, each follow-up time point, and on the off months of each follow-up time point, that is, on the months when a follow-up assessment is not being conducted.

### Statistical Analysis

All primary outcomes are operationalized and assessed in at least two discrete ways, increasing accuracy of measurements, reducing concerns of fully missing data, and allowing for posthoc comparisons of concurrent validity across assessment modalities (Table 1 shows all outcome operationalizations). Descriptive statistics will be calculated and provided for all outcomes, with specific metrics chosen based on the distributional properties of each variable (eg, counts and percentages for nominal variables, means and standard deviations for parametric continuous variables, and ranges and medians for nonparametric continuous variables). All statistical analyses will be carried out using Stata 16SE [85], although the analytical methods described are amenable to most contemporary analytical programs.

**Table 1:** Instruments, targets, variable operationalizations, and minimum detectable effects (1−β=.80; α=.05, 2-tailed).

Instrument	Target	Variable operationalizations				Power-minimum detectable effects		
		Dichotomous	Count	Continuous	Model 1—randomized controlled trial (primary aim 3a; n=150 and n=150)		Model 2—Tx^a^ effects (primary aim 3b; N=300)	Model 3—Matched comparison (primary aim 3c; N=300/ N=~600)
DSM-5^b^ (Meth^c^)	Diagnosis of meth use disorder	Presence or absence of meth use disorder	Number of diagnostic criteria endorsed	N/A^d^	Potential statistical controls; exploratory subgroup analyses		N/A	N/A
URICA^e^	Stage of change or readiness for change	Above or below “Action” stage	N/A	URICA scores	Potential statistical controls; exploratory subgroup analyses		N/A	N/A
Admissions and follow-up form	Sociodemographics; HIV status and Tx history, barriers, and facilitators	Sexual identity; HIV status; linked or unlinked; housing insecurity	Prior drug treatment episodes; symptomology	Age; income	Potential statistical controls; HIV-related subgroup analyses; barrier or facilitator moderator analyses		N/A	N/A
Admissions and follow-up form	HIV prevention or care continuum^f^	HIV test; PrEP^g^ uptake; advance along HIV prevention or care continuum	Number of HIV prevention or care continua steps completed	N/A	Di^h^: OR^i^=1.97 Cu^j^: IRR^k^=1.51		Di: OR=1.61 Cu: IRR=1.35	Di: OR=1.52 Cu: IRR=1.30
Biomarker tests (urinalysis, viral load, dried blood spot, HIV/STI^l^)	Meth use, HIV prevention/care continuum outcomes, sexual risk behavior	Incident STI or incident HIV	Log reductions in HIV VL; DBS analysis of PrEP	Treatment effectiveness score	Variable, dependent on biomarker.		Variable, dependent on biomarker.	Variable, dependent on biomarker.
Substance use frequency	Meth use	Use or nonuse	Days of use	N/A	Di: OR=0.48 Cu: IRR=0.85		Di: OR=0.59 Cu: IRR=0.89	Di: OR=0.62 Cu: IRR=0.90
Behavioral Questionnaire-Amphetamine	HIV sexual risk behavior	CAI^m^	Number of CAI episodes	HIV sexual risk scale	Di: OR=0.52 Cu: IRR=0.78 Co^n^: f^2^^o^=0.03		Di: OR=0.63 Cu: IRR=0.85 Co: f^2^=0.01	Di: OR=0.66 Cu: IRR=0.87 Co: f^2^=0.01
Locator	Contact information	N/A	N/A	N/A	N/A		N/A	N/A

^a^Tx: treatment.

^b^DSM: Diagnostic and Statistical Manual of Mental Disorders-5.

^c^meth: methamphetamine.

^d^NA: not applicable.

^e^URICA: University of Rhode Island Change Assessment.

^f^HIV prevention continuum (HIV testing and pre-exposure prophylaxis or postexposure prophylaxis uptake) and HIV care continuum (link, antiretroviral therapy adherence, and virological suppression).

^g^PrEP: pre-exposure prophylaxis.

^h^Di: dichotomous variable.

^i^OR: odds ratio; estimated 95% CIs are not reported due to the lack of concrete variance estimates to apply to the estimated mean.

^j^Cu: count variable.

^k^IRR: incident rate ratio.

^l^STI: sexually transmitted infection.

^m^CAI: condomless anal intercourse; this includes both receptive and insertive anal intercourse and will be assessed by partner type (eg, main, casual, and exchange).

^n^Co: continuous variable.

^o^f^2^: multiple linear regression effect size estimation.

Diagnostic (ie, DSM-5), psychosocial (eg, URICA), barriers and facilitators (eg, housing insecurity, lack of transportation), and/or sociodemographic variables will be tested for significant association with study outcomes (ie, advancement through the HIV prevention or care continuum, meth use, and HIV sexual risk behaviors), with specific tests of association chosen based on the distributional properties of the outcome variables in question. All variables demonstrating significant statistical association with one or more of the study outcomes will be included as statistical covariates in all multivariate outcome analyses associated with primary aims 3a, 3b, and 3c and will additionally be included in exploratory subgroup analyses to test for moderating effects on treatment response and/or contingent effects among subsets of participants. Primary outcome analyses for primary aims 3a, 3b, and 3c will be carried out using mixed effects generalized linear model (GLM) equations.

Advancement through the HIV prevention or care continuum will be assessed at all time points throughout the study and will be operationalized dichotomously (ie, yes or no achievement of one of the steps on either the HIV prevention or care continuum; eg, viral suppression) and as a counted variable (eg, consecutive DBS results indicating successful PrEP adherence). Power calculations related to advancement through either continuum will assume a 30% probability of achievement of at least one of the steps on either the HIV prevention or care continuum (equivalent to approximately 1:2 odds of advancement). Meth use outcomes will be assessed repeatedly (ie, at all time points) and will be operationalized dichotomously (eg, meth-metabolite-free urine sample) as a counted variable (ie, number of meth-metabolite-free urine samples provided) and as a continuous variable (ie, treatment effectiveness score [86] [total number of meth-metabolite-free samples divided by total samples possible]). Power calculations related to meth use outcomes assumed an 80% probability of meth use during the preintervention period for participants in the DD arm (equivalent to 4:1 odds of use).

HIV sexual risk behavior outcomes will be assessed at all time points throughout the study and will be operationalized dichotomously (eg, incident STI via biomarker testing) as a counted variable (eg, number of days or times engaged in CAI in the past 30 days) and as a continuous variable (eg, an HIV risk severity index generated from multiple factor-analyzed items). Power calculations for engagement in HIV sexual risk behaviors assume a 65% probability of engagement during the preintervention period for participants in the DD arm (equivalent to approximately 2:1 odds of engagement).

Multivariable inferential analyses of dichotomous outcomes will take the form of GLMs employing the Bernoulli family and logit link function; counted outcomes will be analyzed with GLMs employing the Poisson-log or negative binomial link functions, as distributional patterns dictate (note: if after the participant data collection period ends the data evidence an overrepresentation of zeros or an overdispersion of variance, zero-inflated Poisson and/or negative binomial analyses may be substituted). Continuous outcomes will be analyzed using GLMs employing the identity link and Gaussian family functions and assume a single covariate unless otherwise stated (note: iteratively reweighted, bootstrapped, or jackknifed estimation procedures may be used in Gaussian models if sensitivity analyses indicate an undue influence from outliers). Mixed effects GLM models are considered the best linear unbiased estimators for repeated measures data employing nonparametric and/or limited dependent variables. All power calculations are premised on tests of association across 2 time points (eg, baseline with app completion and app completion with brick-and-mortar program completion), providing the most conservative estimate of minimum detectable effect size estimations. All power calculations assume α≤.05 (2-tailed) and 1−β=.80.

## Results

Recruitment began in January 2019 for phase 1, the formative phase. In January and February 2019, 4 separate focus groups (N=36) were conducted to provide input on the adaptation of the Getting Off group-based manual intervention to a cross-platform, computerized mobile app–based intervention. The data collection for phase 2, the RCT, is expected to be completed in January 2023. The final results are anticipated in April 2023.

## Discussion

The Getting Off app is designed to expand access to effective, affordable, private, culturally competent, and highly scalable meth treatment for MSM. MSM experience higher rates of meth use relative to non-MSM, as meth use is deeply integrated into the sexual identities and sexual behaviors among MSM. Use of meth by this population, before or during sex, is associated with decreased behavioral inhibition and increased engagement in HIV risk behaviors. Given the growth of mobile technology, it is no longer reasonable to limit meth treatment options to physical sites, clustered in urban areas and administered using generic, nontailored content. A cross-platform mHealth smartphone app is well suited for engaging MSM because apps are easily accessible, widely used, private, portable, and inexpensive.

There are limitations to the Getting Off study. Given that the study procedures are entirely virtual and that the intervention is app based, enrollment is limited to those that can afford a modern smartphone with an active monthly data plan, as the study does not provide smartphones to participants. However, given that smartphone use is ubiquitous among this population, it is expected that this will not be a major impediment to enrollment. In addition, the study will be conducted in a west coast metropolitan city, and therefore, findings may not be representative of meth-using MSM in other regions of the United States or globally. Finally, the sample may be biased toward MSM who are more likely to enroll in a research study; thus, it may not reflect meth-using MSM that do not self-select to participate in a clinical trial.

The Getting Off app aims to reduce meth use and improve HIV prevention and care continuum outcomes among MSM by providing a culturally competent, user-friendly, and attractive interface that promotes reduced drug use and engagement in high-risk sexual behaviors. The Getting Off mobile app is highly scalable, and if successful, it can be made publicly accessible through the Apple App Store for iOS platforms or Google Play Store for Android platforms for widespread distribution. Given that meth use has also been associated with negative physical and mental health consequences [14,45,50-53], the overall public health benefits from an efficacious meth treatment app could be tremendous.

## Appendix

Multimedia Appendix 1 Peer review report by the NIH Center for Scientific Review.

## Abbreviations

ART: antiretroviral therapy
BQA: Behavioral Questionnaire-Amphetamine
CAI: condomless anal intercourse
CBT: cognitive behavioral therapy
CDC: Centers for Disease Control and Prevention
DBS: dried blood spot
DD: delayed delivery
DSM: Diagnostic and Statistical Manual of Mental Disorders
GLM: generalized linear model
ID: immediate delivery
IRB: Institutional Review Board
meth: methamphetamine
mHealth: mobile health
MSM: men who have sex with men
PEP: postexposure prophylaxis
PrEP: pre-exposure prophylaxis
SOC: stages of change
STI: sexually transmitted infection
URICA: University of Rhode Island Change Assessment
