# Supplementary material for: A Theory-Based mHealth Intervention (Getting Off) for Methamphetamine-Using Men Who Have Sex With Men: Protocol for a Randomized Controlled Trial
Source: JMIR Res Protoc. 2021 Feb 22;10(2):e22572. doi: 10.2196/22572 (PMC7939940; doi:10.2196/22572)
Supplement: Multimedia Appendix 1 [file resprot_v10i2e22572_app1.pdf]

SUMMARY STATEMENT
(Privileged Communication)

PROGRAM CONTACT:
Guifang Lao
301-443-1061
laog@mail.nih.gov

Release Date: **03/26/2018**
Revised Date:

Application Number: **1 R01 DA045562-01A1**

Principal Investigator

REBACK, CATHY J

Applicant Organization: FRIENDS RESEARCH INSTITUTE, INC.

Review Group: **BSPH**
Behavioral and Social Science Approaches to Preventing HIV/AIDS Study Section

Meeting Date: **03/08/2018**
Council: **MAY 2018**
Requested Start: **07/01/2018**
RFA/PA: **PA16-072**
PCC: **CC/GLA**

Project Title: **Getting Off: A Theory-based mHealth Intervention for Methamphetamine-using MSM**
SRG Action: **Impact Score:32 Percentile:13**
Next Steps: **Visit https://grants.nih.gov/grants/next_steps.htm**
Human Subjects: 30-Human subjects involved - Certified, no SRG concerns
Animal Subjects: 10-No live vertebrate animals involved for competing appl.
Gender: 3A-Only men, scientifically acceptable
Minority: 1A-Minorities and non-minorities, scientifically acceptable
Children: 3A-No children included, scientifically acceptable

Project Year	Direct Costs Requested	Estimated Total Cost
1	405,197	498,588
2	383,635	472,056
3	433,028	532,834
4	499,998	615,239
5	391,450	481,673
TOTAL	2,113,308	2,600,390

ADMINISTRATIVE BUDGET NOTE: The budget shown is the requested budget and has not been adjusted to reflect any recommendations made by reviewers. If an award is planned, the costs will be calculated by Institute grants management staff based on the recommendations outlined below in the COMMITTEE BUDGET RECOMMENDATIONS section.

1R01DA045562-01A1 Reback, Cathy

RESUME AND SUMMARY OF DISCUSSION: The applicant proposes to continue the development of a mobile phone based app and to test it among methamphetamine (meth) using MSM. Smartphones are routinely used by MSM to seek out sex partners; moreover, meth use in that group has been associated with higher rates of HIV transmission and prevalence. Therefore, designing "Getting Off," the app designed to reduce meth use and HIV risk behaviors preliminarily testing it marries these two phenomena well. The premise of the application is evidently very strong given that MSM are the most at-risk group for HIV infection. The investigators are an outstanding group of collaborators with the requisite training and experience to address every critical component of the application. The application is very appropriately grounded in two theories: Cognitive Behavioral theory and the Stages of Change model. This resubmission was very responsive to the prior review; among its many improvements, the applicants have abandoned internet recruitment which would have made it difficult to verify specific demographics of the participants and be able to administer biomarkers. They will now sample their participants from Los Angeles County and will ascertain that they are at least 18 years old. In addition, dried blood spots analyses will be conducted to determine PrEP uptake and adherence and urinalyses to ascertain drug use. Applicants have also provided preliminary evidence that users found the app engaging. Overall, the application is very well crafted; the methods and analyses are generally very robust and the applicants have accounted for sex as a biological variable. There were however some minor weaknesses and differences of opinion which very slightly dampened enthusiasm. The focus on MA use seems to ignore that Black MSM, who are among the most vulnerable are not at highest risk for using MA; moreover, age range is rather broad as an inclusion criterion; and age may account for differential use of the apps and could be a confounding variable unless accounted for; it is also unclear whether 24 sessions of the app may not represent a burden for the participants. Finally, reviewers disagree that the approach was somewhat weakened because the preliminary data support acceptability of the app but not whether or not it influences behavior. Other reviewers thought that the preliminary measures of efficacy were sufficiently strong to make the point. Despite these issues, most of the reviewers were impressed by the careful development and robustness of the application which they feel will have a very high impact of the health of meth-using MSM.

DESCRIPTION (provided by applicant): Methamphetamine (MA) use among men who have sex with men (MSM) is associated with increased rates of HIV prevalence and transmission, as well as substandard advancement along the HIV Prevention and Care Continua. MA use among MSM is deeply integrated into socio-sexual networks including the use of smartphone applications ("app") and websites to find sexual partners. Given the growth of mobile health technology, it is no longer necessary or reasonable to limit MA treatment options to physical sites, clustered in urban areas, and administered using generic, non-tailored content. The project builds upon the established efficacy of our manualized MA-abuse treatment intervention, "Getting Off: A Behavioral Treatment Intervention for Gay and Bisexual Male Methamphetamine Users," and the highly promising findings from our successful Stage I proof-of-concept study, to complete translation of Getting Off into a cross-platform (iOS and Android) app and assess the app's efficacy and non-inferiority in a scientifically rigorous randomized trial. The Getting Off app, like the group-based intervention before it, will use the principles of Cognitive Behavioral Theory and Stages of Change to help MSM reduce or eliminate MA use and HIV sexual risk behaviors, and increase advancement along the HIV Prevention or Care Continuum (including uptake of HIV testing, pre-, and post- exposure prophylaxis [PEP/PrEP] and PrEP adherence and persistence for those who are HIV negative; ART uptake and adherence for those who are HIV positive). This project will 1) refine and enhance the first 8 sessions of the Getting Off MA-abuse treatment intervention developed in Stage I based on feasibility pilot test user feedback, 2) conduct formative research to develop the remaining 16 sessions of the Getting Off MA- abuse treatment intervention into a cross-platform computerized mobile app targeted to reduce MA use and HIV sexual risk behaviors, and increase advancement along the HIV Prevention or Care Continuum, and 3) conduct a RCT to evaluate reductions of MA use and HIV sexual risk behaviors, and increased advancement along the

HIV Prevention or Care Continuum, using three approaches: a) Efficacy Trial – a two-arm RCT to determine intervention effects through comparison of the Immediate Delivery (ID; n=150) and Delayed Delivery (DD; n=150) arms; b) Efficacy Trial – an observed treatment effects analysis to compare pre/post data from the pooled ID and DD conditions (N=300); and, c) Non-inferiority Trial – a two-arm historical matched comparison design to evaluate the outcomes of the Getting Off app (ID + DD; N=300) relative to a matched sample of participants having previously attended the brick-and-mortar group-based Getting Off intervention (N~600; total N=900). The RCT uses repeated measures to assess participants at baseline, 1-, 2- (DD condition only), 3-, 6-, and 9-month follow-up. This study could have significant public health impact by greatly expanding access to effective, affordable, private, culturally competent and highly scalable MA treatment to this very high-risk population.

PUBLIC HEALTH RELEVANCE: Methamphetamine (MA) use among MSM is strongly associated with HIV infection and interrupted progression along the HIV Prevention and Care Continua. This study will complete the development and evaluation of an evidence-based, theory-driven, and culturally competent treatment app for MA-using MSM, designed to reduce or eliminate MA use and HIV sexual risks, increase uptake of HIV testing and pre- and post-exposure prophylaxis (PrEP/PEP), including PrEP adherence and persistence for those who are HIV negative, and increase retention in HIV care and adherence to ART for those who are HIV positive. Given the severe personal and public health consequences of MA use, the public health significance of this app is very high as it will provide this population with a tailored treatment opportunity that is easily accessible, affordable, private, and highly scalable.

CRITIQUE 1

Significance: 1
Investigator(s): 1
Innovation: 1
Approach: 2
Environment: 1

Overall Impact: Proposed is a multi-phased intervention development and non-inferiority trial focused on the adaption to mobile phone of an evidenced-based, group, cognitive behavioral theory and stages of change intervention to reduce methamphetamine use and HIV risk for men who have sex with men (MSM) entitled “Getting off.” This revised application has been improved with the inclusion of biomarkers for HIV, STIs, and PrEP adherence, inclusion of alpha-beta testing of the remaining intervention adaption components (16 of 24 total modules), use of back-end data to assess user engagement with the application, focus on Los Angeles County that better matches available historic control group (that received the intervention live and in a group setting), and additional pilot information that indicates acceptability and feasibility of app use by MSM. The scientific premise for this study is outstanding. MSM are documented to use smartphone applications for a number of purposes and as an intervention tool, acceptability of this approach has been proven. The historic comparison group makes sense (the non-inferiority trial) as the intervention was efficacious in the original format. The investigative team is very strong and has an outstanding record of accomplishment. The approach is feasible and the study design is rigorous. HIV continues to be a challenge among MSM and methamphetamine use in combination with unsafe sex is one reason the HIV infection remain so high in this population. The rigorous testing of an intervention for smartphones that might reduce both methamphetamine use and HIV risk is greatly needed. Some minor issues in approach were identified. The proposed research is highly significant and innovative. The environment is well suited to support this research. Enthusiasm for the proposed research is very high.

1. Significance:

Strengths

- MSM remain at elevated risk for HIV and STI transmission.
- Methamphetamine use is a known risk factor for HIV risk and transmission among MSM.
- MSM have been found to be receptive to mobile phone-based information and interventions.
- The adaption of on-ground, evidence-based interventions for use by MSM via mobile phones is of high public health significance.

Weaknesses

- None Noted

2. Investigator(s):

Strengths

- The investigative team is exceptionally well-prepared to conduct this research.

Weaknesses

- None Noted

3. Innovation:

Strengths

- Making proven interventions usable via smartphone technology is innovative.
- Cultural and individual personalization of the app is innovative and likely to make it more appealing to at-risk populations.

Weaknesses

- None Noted

4. Approach:

Strengths

- Appropriate use of formative research methods to complete adaption of the on-ground, evidence-based intervention
- Alpha-beta testing of complete app is a strength
- Use of historic control is appropriate within the context of the non-inferiority trial.
- Measures are all reliable and valid.
- Use of back-end data to examine user engagement is good.
- Highly feasible research project.

Weaknesses

- Broad range of participants may be a challenge if age differences in response to the intervention occur. Seems like this would be likely given generational differences in technology use and comfort.

5. Environment:

Strengths

- Excellent

Weaknesses

- None Noted

Protections for Human Subjects:

Acceptable Risks and/or Adequate Protections

- Procedures for the protection of human subjects are adequate.

Data and Safety Monitoring Plan (Applicable for Clinical Trials Only):

Acceptable

- Data safety plan is appropriate.

Inclusion of Women, Minorities and Children:

- Sex/Gender: Distribution justified scientifically
- Race/Ethnicity: Distribution justified scientifically
- For NIH-Defined Phase III trials, Plans for valid design and analysis: Scientifically acceptable
- Inclusion/Exclusion of Children under 18: Excluding ages <18; justified scientifically
- Justification for gender and racial characteristics of sample are appropriate.

Vertebrate Animals:

Not Applicable (No Vertebrate Animals)

Biohazards:

Acceptable

Resubmission:

- This revised application has been very responsive to prior reviewer comments.

Authentication of Key Biological and/or Chemical Resources:

Not Applicable (No Relevant Resources)

Budget and Period of Support:

Recommend as Requested:

CRITIQUE 2

Significance: 2
Investigator(s): 1
Innovation: 3

Approach: 3
Environment: 1

Overall Impact: In this proposal the investigators aim to refine, pilot and implement an app to improve the health and well-being of methamphetamine using MSM. The study design is rigorous with several iterations in app development as well as multiple testing and refinement pieces all built upon pilot data. The RCT also includes historical cohort which adds a low cost new dimension to the analysis. The team has several leading experts in the areas of app development, meth using MSM, and PrEP. There were several minor weaknesses which all seem addressable.

1. Significance:

Strengths

- Meth using MSM are at increased risk of HIV and onwards transmission.

Weaknesses

- Young Black MSM are not at highest risk of MA use

2. Investigator(s):

Strengths

- Strong team of accomplished investigators

Weaknesses

- None Noted

3. Innovation:

Strengths

- An app for meth addiction would be highly innovative

Weaknesses

- Most of the innovations described are for apps broadly speaking and not “Getting Off.”

4. Approach:

Strengths

- CBT and stages of change description and process is very clear
- Very clear process for testing and retesting
- Use of second control group (historical) is useful and low cost

Weaknesses

- 22 sessions seem like a lot and unclear how self-direction will occur
- Inclusion criteria for age is broad swath; would think about focusing on narrower age range and particularly younger MSM
- Split between bringing recruit to site and enrollment is unnecessary
- If the Getting Off in person intervention is being used and offered through health department funding; why not use control as real time instead of historical control?

- Why fingerstick and phlebotomy? Why not just test for HIV with 4th generation of phlebotomy?
- Four viral load and PrEP adherence outcomes; what will be done if they have baseline adherence? May decrease power and probably should only include those with poor adherence.
- Power for aim 3a is for sexual behavior and not care continuum outcomes
- Why are structural factors treated as moderating variables and not primary outcome variables?
- Biomedical prevention could be better developed.

5. Environment:

Strengths

- Longstanding supportive environment for drug use research

Weaknesses

- None Noted

Protections for Human Subjects:

Acceptable Risks and/or Adequate Protections

Data and Safety Monitoring Plan (Applicable for Clinical Trials Only):

Acceptable

Inclusion of Women, Minorities and Children:

- Sex/Gender: Distribution justified scientifically
- Race/Ethnicity: Distribution justified scientifically
- For NIH-Defined Phase III trials, Plans for valid design and analysis:
- Inclusion/Exclusion of Children under 18: Excluding ages <18; justified scientifically

Vertebrate Animals:

Not Applicable (No Vertebrate Animals)

Biohazards:

Not Applicable (No Biohazards)

Budget and Period of Support:

Recommend as Requested:

CRITIQUE 3

Significance: 3
Investigator(s): 1
Innovation: 2

Approach: 4
Environment: 3

Overall Impact: This is a very strong resubmitted application led by an excellent team. The premise of this application is valid and strong and is that reducing meth use among MSM will contribute to reduced HIV risk among them, and that since many MSM use mobile devices to seek out sexual encounters, mHealth applications of meth-use treatment may be an affordable and accessible mechanism to deliver the intervention and have an impact on their risk-taking behaviors. There are many strengths to this application. The PI is outstanding and the team she has put together including the app development group are well qualified and capable of carrying out the proposed research. The intervention is a digitalized app that is based on an evidence-based brick-and-mortar meth-treatment program targeting MSM. If what the investigators purport to do in this application comes to pass, it is likely to have at least some impact on risk-taking behaviors among MSM. It is also highly innovative. The intervention is premised on Cognitive Behavioral Therapy and the Stages of Change model. The application is innovative in that it digitizes these two powerful and established mechanisms of behavior change. The methodological approach overall is for the most part rigorous and transparent. It has many strengths, including the use of urine-testing for substance use and viral load testing as an indicator of engagement in the HIV treatment continuum for those who are HIV-infected. In most respects the investigators have been responsive to previous reviews but there are two particularly important exceptions which were the biggest factors influencing the overall impact score. First, they demonstrate acceptability in the innovation and approach sections of an app for a health-centered intervention in MSM but have no preliminary data about whether or not it gets actually used or has an impact. What will the investigators do if they find that for some reason the app is not feasible? The efficacy is in part addressed with a non-inferiority efficacy trial, but the application would be greatly strengthened if they had some preliminary data to demonstrate that it actually does have an impact on meth-use and/or HIV risk behaviors. Second, the majority of the digital sessions still need to be created as a condition of trialability. The investigators provide convincing evidence in the preliminary studies section that they can do this translation based on the first sessions they have piloted, but there is a lot of formative work to be done that might preclude or prevent the clinical trial from moving forward.

1. Significance:

Strengths

- Meth use is a serious and prevalent issue among MSM in North America. It is contributing significantly to the ongoing HIV epidemic among this population.
- The premise of this application is valid and is that reducing meth use among MSM will contribute to reduced HIV risk among them, and that since many MSM use mobile devices to seek out sexual encounters, mHealth applications of meth-use treatment may be an affordable and accessible mechanism to deliver the intervention and have an impact on their risk-taking behaviors.
- If what the investigators purport to do in this application comes to pass, it is likely to have at least some impact on risk-taking behaviors among MSM.

Weaknesses

- Although MSM use mobile devices for a plethora of pleasure-seeking reasons, the significance section does not present evidence that it would equally have an impact on health promotion/wellness seeking behavior such as reduced meth use.

2. Investigator(s):

Strengths

- The PI is a senior research scientist with Friends Research Institute and a highly accomplished investigator. She also serves as the Core Director of the Center for HIV Identification, Prevention, and Treatment Services at UCLA. Her major contributions to science are related to methamphetamine use and HIV risk in MSM, combination HIV prevention particularly related to substance use reduction, the application of research in community-based settings, and research with transgender women and gender non-conforming individuals. She is the PI of several federally funded studies of relevance to this application, and a co-investigator on several others.
- The co-investigators and consultants listed on the application are all highly qualified experts in their fields with strong research track records.

Weaknesses

- None Noted

3. Innovation:

Strengths

- The intervention app is highly innovative. It ‘gamifies’ a former group-based intervention.
- The app will include self-administered ‘risk calculators’ and an all-in-one product containing functionality that MSM desire in a health promotion app.
- The app can be used any time of day and as the investigators point out can be used by MSM in the evening or late at night when brick-and-mortar sites are typically closed so if they are having a craving they can access support in real time when risk is greatest.
- The intervention is premised on Cognitive Behavioral Therapy and the Stages of Change model. The application is innovative in that it digitizes these two powerful and established mechanisms of behavior change.
- The ‘back-end’ data that can be collected from this research could be extremely useful in informing treatment providers of the facilitators most relevant to MSM.

Weaknesses

- None Noted

4. Approach:

Strengths

- The application is based on an intervention that in a brick-and-mortar setting has been experimentally proven to be highly effective.
- Proof of concept of the app has been demonstrated and disseminated.
- Radiant Creative Group has been selected as the app development experts. Co-investigator Horvath has worked successfully with them on two previous mobile apps. They provide the necessary technical expertise, creativity and skill necessary to carry-out this study.
- The investigators explicitly are proposing a cross-platform framework (iOS and Android).
- The app (vs. brick-and-mortar) delivery is more likely to engage people who have at least moved beyond the precontemplation stage of change, where the brick-and-mortar delivery is more likely to engage individuals who are already in the ‘action’ stage by the time they seek treatment.
- A major strength of the app vs. brick-and-mortar delivery is that individuals do not always progress in a linear fashion through the stages of change but go back over and over until they

are ready to move forward. The app allows them to make repeated attempts over time if/when they relapse.

- Urine drug screening will confirm substance use.
- The power calculations and analysis plan are generally robust. The investigators might consider using a state-space modeling framework to evaluate progression through the cascade (Lee et al. Stats Med 2017)
- Sex as a biological variable – Men only will be included in the study and this is warranted given the target population. The investigators are to be commended on including transgender masculine individuals who identify as either gay or bisexual (but not lesbian) and identify as a man who has sex with men.

Weaknesses

- There is an inconsistency in the order of randomization and baseline data collection – in the text it says the baseline data collection will happen prior to randomization but in Figure 2 it happens after. Because the arm assignment is not blinded, the order of these procedures actually could matter.
- What is missing from the power calculation and analysis plan is adjustment for intra-individual clustering, multiple comparisons likely requiring a Bonferonni or equivalent adjustment method, and any adjustment for non-independence between individuals (especially if recruitment is partially using snowball-sampling for the RCT it is likely that the individuals who enroll knowing each other will offer mutual support in reducing substance use and risky sexual behavior.
- The alternative strategies plan is a limitation. What will the investigators do if they find that for some reason the app is not feasible? The efficacy is in part addressed with a non-inferiority efficacy trial, but the application would be greatly strengthened if they had some preliminary data to demonstrate that it actually does have an impact on meth-use and/or HIV risk behaviors. The majority of the digital sessions still needs to be created as a condition of trialability. The investigators provide convincing evidence in the preliminary studies section that they can be based on the first sessions they have piloted, but there is a lot of formative work to be done that might preclude or prevent the clinical trial from moving forward.

5. Environment:

Strengths

- The Friends Research Institute is a well-established non-profit research institution. They have adequate infrastructure to enable the successful implementation of the study specifically related to personnel and computing and communication capabilities, as do the other participating institutions.
- The actual site for the study is the Friends Community Center, a division of FRI, which the PI established and is a community-based outreach center where a lot of the research undertaken by the institute takes place as well as various service programs. It has ample splash, a CLIA-certified laboratory linked to the UCLA Care Center and has the capacity to conduct all the point-of-care screening for the study.

Weaknesses

- None Noted

Protections for Human Subjects:

Acceptable Risks and/or Adequate Protections

- All necessary categories in this section have been addressed appropriately. There are no unnecessary risks for participants in this study and acceptable measures are in place to protect participants against harm.

Data and Safety Monitoring Plan (Applicable for Clinical Trials Only):

Acceptable

- This study meets the criteria for a clinical trial. They have outlined a very reasonable data and safety monitoring plan, including using the DSMB of UCLA Dept. of Family Medicine. One member of the DSMB will be the Medical Safety Officer for the study who will assist the PI to evaluate whether an active subject should be discontinued for safety reasons.

Inclusion of Women, Minorities and Children:

- Sex/Gender: Distribution justified scientifically
- Race/Ethnicity: Distribution justified scientifically
- For NIH-Defined Phase III trials, Plans for valid design and analysis: Not applicable
- Inclusion/Exclusion of Children under 18: Excluding ages <18; justified scientifically
- Only males will be eligible to participate in this study, which is appropriate given the study aims. Approximately two-thirds of participants are expected to be non-Caucasian, also appropriate given the epidemiology of meth-use among MSM. Individuals under the age of 18 are excluded which is appropriate since there is no obvious need to include children. The age range of the proposed study mirrors the age eligibility criterion of the three prior studies that were conducted on the original intervention.

Vertebrate Animals:

Not Applicable (No Vertebrate Animals)

Biohazards:

Not Applicable (No Biohazards)

Resubmission:

- Most of the major comments raised by previous reviewers have been addressed, notably the incorporation of urine testing and viral load testing to measure substance use and for those living with HIV, where they are on the treatment continuum (granted viral load is only one measure, but it is really the one that matters.)
- Some of the limitations remain, most of which are relatively minor. Two that stand out are 1) One assumes but it is not clearly stated that the reason for two arms of immediate delivery vs. delayed delivery is because of the potential effect of temporal trends on outcomes of interest. Thirty days is not a lot of time to affect temporal trends. 2) There is still not an inclusion criterion around having a smartphone or tablet and access to the internet.
- The major ones that remain unaddressed are 1) that while the investigators have demonstrated acceptability in pilot work, even preliminary feasibility and efficacy have not. What will the investigators do if they find that for some reason the app is not feasible? The efficacy is in part addressed with a non-inferiority efficacy trial, but the application would be greatly strengthened if they had some preliminary data to demonstrate that it actually does have an impact on meth-use and/or HIV risk behaviors. 2) The majority of the digital sessions still need to be created as

a condition of trialability. The investigators provide convincing evidence in the preliminary studies section that they can be based on the first sessions they have piloted, but there is a lot of formative work to be done that might preclude or prevent the clinical trial from moving forward.

Resource Sharing Plans:

Unacceptable

- There is no discussion of this. The investigators are investing considerable resources in the app which will be a resource with a lot of potential (if the study is successful). Are they planning on sharing it? Selling it? Giving it away?

Authentication of Key Biological and/or Chemical Resources:

Not Applicable (No Relevant Resources)

Budget and Period of Support:

Recommend as Requested:

THE FOLLOWING SECTIONS WERE PREPARED BY THE SCIENTIFIC REVIEW OFFICER TO SUMMARIZE THE OUTCOME OF DISCUSSIONS OF THE REVIEW COMMITTEE, OR REVIEWERS' WRITTEN CRITIQUES, ON THE FOLLOWING ISSUES:

PROTECTION OF HUMAN SUBJECTS: ACCEPTABLE

INCLUSION OF WOMEN PLAN (G3A): ACCEPTABLE

INCLUSION OF MINORITIES PLAN (M1A): ACCEPTABLE

INCLUSION OF CHILDREN PLAN (C3A): ACCEPTABLE

COMMITTEE BUDGET RECOMMENDATIONS: The budget was recommended as requested.

Footnotes for 1 R01 DA045562-01A1; PI Name: Reback, Cathy J

NIH has modified its policy regarding the receipt of resubmissions (amended applications). See Guide Notice NOT-OD-14-074 at http://grants.nih.gov/grants/guide/notice-files/NOT-OD-14-074.html. The impact/priority score is calculated after discussion of an application by averaging the overall scores (1-9) given by all voting reviewers on the committee and multiplying by 10. The criterion scores are submitted prior to the meeting by the individual reviewers assigned to an application, and are not discussed specifically at the review meeting or calculated into the overall impact score. Some applications also receive a percentile ranking. For details on the review process, see http://grants.nih.gov/grants/peer_review_process.htm#scoring.

MEETING ROSTER

Behavioral and Social Science Approaches to Preventing HIV/AIDS Study Section
AIDS and Related Research Integrated Review Group
CENTER FOR SCIENTIFIC REVIEW
BSPH
03/08/2018 - 03/09/2018

Notice of NIH Policy to All Applicants: Meeting rosters are provided for information purposes only. Applicant investigators and institutional officials must not communicate directly with study section members about an application before or after the review. Failure to observe this policy will create a serious breach of integrity in the peer review process, and may lead to actions outlined in NOT-OD-14-073 at https://grants.nih.gov/grants/guide/notice-files/NOT-OD-14-073.html and NOT-OD-15-106 at https://grants.nih.gov/grants/guide/notice-files/NOT-OD-15-106.html, including removal of the application from immediate review.

CHAIRPERSON(S)

ROSSER, B R SIMON, MPH, PHD
PROFESSOR
DIVISION OF EPIDEMIOLOGY AND COMMUNITY HEALTH
SCHOOL OF PUBLIC HEALTH
UNIVERSITY OF MINNESOTA
MINNEAPOLIS, MN 55454

MEMBERS

ALLEN, SUSAN A, MD, MPH
PROFESSOR
DEPARTMENT OF PATHOLOGY
AND LABORATORY MEDICINE
SCHOOL OF MEDICINE
EMORY UNIVERSITY
ATLANTA, GA 30322

BANKOLE, AKINRINOLA, PHD *
DIRECTOR
INTERNATIONAL RESEARCH
THE GUTTMACHER INSTITUTE
NEW YORK, NY 10005

BLANKENSHIP, KIM M, PHD *
PROFESSOR AND CHAIR
DEPARTMENT OF SOCIOLOGY
AMERICAN UNIVERSITY
WASHINGTON, DC 20016

BLUTHENTHAL, RICKY N, PHD
PROFESSOR
DEPARTMENT OF PREVENTIVE MEDICINE
KECK SCHOOL OF MEDICINE
UNIVERSITY OF SOUTHERN CALIFORNIA
LOS ANGELES, CA 90033

BRAITSTEIN, PAULA KARINA ALICE, PHD *
ASSOCIATE PROFESSOR
DEPARTMENT OF OBSTETRICS AND GYNECOLOGY
DALLA LANA SCHOOL OF PUBLIC HEALTH
UNIVERSITY OF TORONTO
TORONTO, ON
CANADA

CASSELS, SUSAN LYNN, PHD *
ASSISTANT PROFESSOR
DEPARTMENT OF GEOGRAPHY
UNIVERSITY OF CALIFORNIA, SANTA BARBARA
SANTA BARBARA, CA 93106

CHARLEBOIS, EDWIN DUNCAN III, PHD, MPH
PROFESSOR
DEPARTMENT OF MEDICINE
SCHOOL OF MEDICINE
UNIVERSITY OF CALFORNIA, SAN FRANCISCO
SAN FRANCISCO , CA 94105

CHRISTOPOULOS, KATERINA A, MD *
ASSOCIATE PROFESSOR
HIV/AIDS DIVISION
SAN FRANCISCO GENERAL HOSPITAL
UNIVERSITY OF CALIFORNIA, SAN FRANCISCO
SAN FRANCISCO, CA 94110

DEGRUTTOLA, VICTOR GERARD, DSC *
PROFESSOR
DEPARTMENT OF BIOSTATISTICS
SCHOOL OF PUBLIC HEALTH
HARVARD UNIVERSITY
BOSTON, MA 02115

EATON, LISA A, PHD *
ASSOCIATE PROFESSOR
DEPARTMENT OF HUMAN DEVELOPMENT
AND FAMILY STUDIES
UNIVERSITY OF CONNECTICUT
STORRS, CT 06029

FUJIMOTO, KAYO, PHD *
ASSOCIATE PROFESSOR
DEPARTMENT OF HEALTH PROMOTIONS
AND BEHAVIORAL SCIENCES
SCHOOL OF PUBLIC HEALTH
UNIVERSITY OF TEXAS AT HOUSTON
HOUSTON, TX 77030

GOGGIN, KATHY J, PHD *
GLASSCOCK CHAIR AND PROFESSOR
HEALTH SERVICES AND OUTCOMES RESEARCH
CHILDREN'S MERCY HOSPITAL AND CLINICS
UNIVERSITY OF MISSOURI, KANSAS CITY
KANSAS CITY, MO 64110

HAVENS, JENNIFER R, PHD, MPH
ASSOCIATE PROFESSOR
DEPARTMENT OF BEHAVIORAL SCIENCE
COLLEGE OF MEDICINE
UNIVERSITY OF KENTUCKY
LEXINGTON, KY 40504

HIGHTOW-WEIDMAN, LISA B, MD, MPH
ASSOCIATE PROFESSOR
DIVISION OF INFECTIOUS DISEASES
DEPARTMENT OF MEDICINE
SCHOOL OF MEDICINE
UNIVERSITY OF NORTH CAROLINA
CHAPEL HILL, NC 27599-7030

KERSHAW, TRACE S, PHD
PROFESSOR
CENTER FOR INTERDISCIPLINARY RESEARCH ON AIDS
DEPARTMENT OF EPIDEMIOLOGY
SCHOOL OF PUBLIC HEALTH
YALE UNIVERSITY
NEW HAVEN, CT 06510

KURTZ, STEVEN P, PHD *
PROFESSOR AND DIRECTOR
CENTER FOR APPLIED RESEARCH ON SUBSTANCE USE
AND HEALTH DISPARITIES
DEPARTMENT OF JUSTICE AND HUMAN SERVICES
NOVA SOUTHEASTERN UNIVERSITY
CORAL GABLES, FL 33134

LOUE, SANA, PHD, JD *
PROFESSOR
CENTER FOR MINORITY HEALTH
DEPARTMENT OF EPIDEMIOLOGY AND BIOSTATISTICS
SCHOOL OF MEDICINE
CASE WESTERN RESERVE UNIVERSITY
CLEVELAND, OH 44106

MACQUEEN, KATHLEEN M, PHD
SENIOR SCIENTIST
SOCIAL AND BEHAVIORAL HEALTH SCIENCES
FHI 360
DURHAM, NC 27701

MORRIS, WANDA MARTINA, PHD *
PROFESSOR
DEPARTMENTS OF SOCIOLOGY AND STATISTICS
UNIVERSITY OF WASHINGTON
SEATTLE, WA 98195

PAUL, ROBERT H, PHD *
PROFESSOR AND DIRECTOR
DEPARTMENT OF PSYCHOLOGICAL SCIENCES
MISSOURI INSTITUTE OF MENTAL HEALTH
UNIVERSITY OF MISSOURI, ST LOUIS
ST LOUIS, MO 02906

RICKS, JANELLE, DRPH *
ASSISTANT PROFESSOR
HEALTH BEHAVIOR AND HEALTH PROMOTION
COLLEGE OF PUBLIC HEALTH
OHIO STATE UNIVERSITY
COLUMBUS, OH 43210

SCHNEIDER, JOHN, MD, MPH
ASSOCIATE PROFESSOR
SECTION OF INFECTIOUS DISEASE AND GLOBAL HEALTH
DEPARTMENT OF MEDICINE
UNIVERSITY OF CHICAGO
CHICAGO, IL 60637

SHERMAN, SUSAN GAIL, PHD
PROFESSOR
DEPARTMENT OF HEALTH, BEHAVIOR, AND SOCIETY
SCHOOL OF PUBLIC HEALTH
JOHNS HOPKINS UNIVERSITY
BALTIMORE, MD 21205

SIMONI, JANE MARIE, PHD
PROFESSOR
DEPARTMENT OF PSYCHOLOGY
UNIVERSITY OF WASHINGTON
SEATTLE, WA 98195

SWEAT, MICHAEL D, PHD
PROFESSOR
DEPARTMENT OF PSYCHIATRY
AND BEHAVIORAL SCIENCES
MEDICAL UNIVERSITY OF SOUTH CAROLINA
CHARLESTON, SC 29407

WENZEL, SUZANNE L, PHD
PROFESSOR AND CHAIR
DEPARTMENT OF ADULT MENTAL HEALTH AND WELLNESS
SCHOOL OF SOCIAL WORK
UNIVERSITY OF SOUTHERN CALIFORNIA
LOS ANGELES, CA 90089

YOUNG, SEAN, PHD *
ASSOCIATE PROFESSOR AND EXECUTIVE DIRECTOR
DEPARTMENT OF FAMILY MEDICINE
UC INSTITUTE FOR PREDICTION TECHNOLOGY (UCIPT)
UNIVERSITY OF CALIFORNIA, LOS ANGELES
LOS ANGELES, CA 90024

ZEA, MARIA CECILIA, PHD *
PROFESSOR
DEPARTMENT OF PSYCHOLOGY
COLLEGE OF ARTS AND SCIENCES
GEORGE WASHINGTON UNIVERSITY
WASHINGTON, DC 20052

SCIENTIFIC REVIEW OFFICER

GUERRIER, JOSE H, PHD
SCIENTIFIC REVIEW OFFICER
CENTER FOR SCIENTIFIC REVIEW
NATIONAL INSTITUTES OF HEALTH
BETHESDA, MD 20892

EXTRAMURAL SUPPORT ASSISTANT

STROTHERS, DIARA
EXTRAMURAL SUPPORT ASSISTANT
CENTER FOR SCIENTIFIC REVIEW
NATIONAL INSTITUTES OF HEALTH
BETHESDA, MD 20892

* Temporary Member. For grant applications, temporary members may participate in the entire meeting or may review only selected applications as needed.

Consultants are required to absent themselves from the room during the review of any application if their presence would constitute or appear to constitute a conflict of interest.
